# Optimization of AAV vectors to target persistent viral reservoirs

**DOI:** 10.1186/s12985-021-01555-7

**Published:** 2021-04-23

**Authors:** Rossana Colón-Thillet, Keith R. Jerome, Daniel Stone

**Affiliations:** 1 Vaccine and Infectious Disease Division, Fred Hutchinson Cancer Research Center, 1100 Fairview Ave N, Seattle, WA USA; 2 Department of Laboratory Medicine, University of Washington, Seattle, WA USA

**Keywords:** Adeno-associated virus, Herpes simplex virus, Hepatitis B virus, Human immunodeficiency virus

## Abstract

Gene delivery of antiviral therapeutics to anatomical sites where viruses accumulate and persist is a promising approach for the next generation of antiviral therapies. Recombinant adeno-associated viruses (AAV) are one of the leading vectors for gene therapy applications that deliver gene-editing enzymes, antibodies, and RNA interference molecules to eliminate viral reservoirs that fuel persistent infections. As long-lived viral DNA within specific cellular reservoirs is responsible for persistent hepatitis B virus, Herpes simplex virus, and human immunodeficiency virus infections, the discovery of AAV vectors with strong tropism for hepatocytes, sensory neurons and T cells, respectively, is of particular interest. Identification of natural isolates from various tissues in humans and non-human primates has generated an extensive catalog of AAV vectors with diverse tropisms and transduction efficiencies, which has been further expanded through molecular genetic approaches. The AAV capsid protein, which forms the virions' outer shell, is the primary determinant of tissue tropism, transduction efficiency, and immunogenicity. Thus, over the past few decades, extensive efforts to optimize AAV vectors for gene therapy applications have focused on capsid engineering with approaches such as directed evolution and rational design. These approaches are being used to identify variants with improved transduction efficiencies, alternate tropisms, reduced sequestration in non-target organs, and reduced immunogenicity, and have produced AAV capsids that are currently under evaluation in pre-clinical and clinical trials. This review will summarize the most recent strategies to identify AAV vectors with enhanced tropism and transduction in cell types that harbor viral reservoirs.

## Introduction

**Fig. 1:**
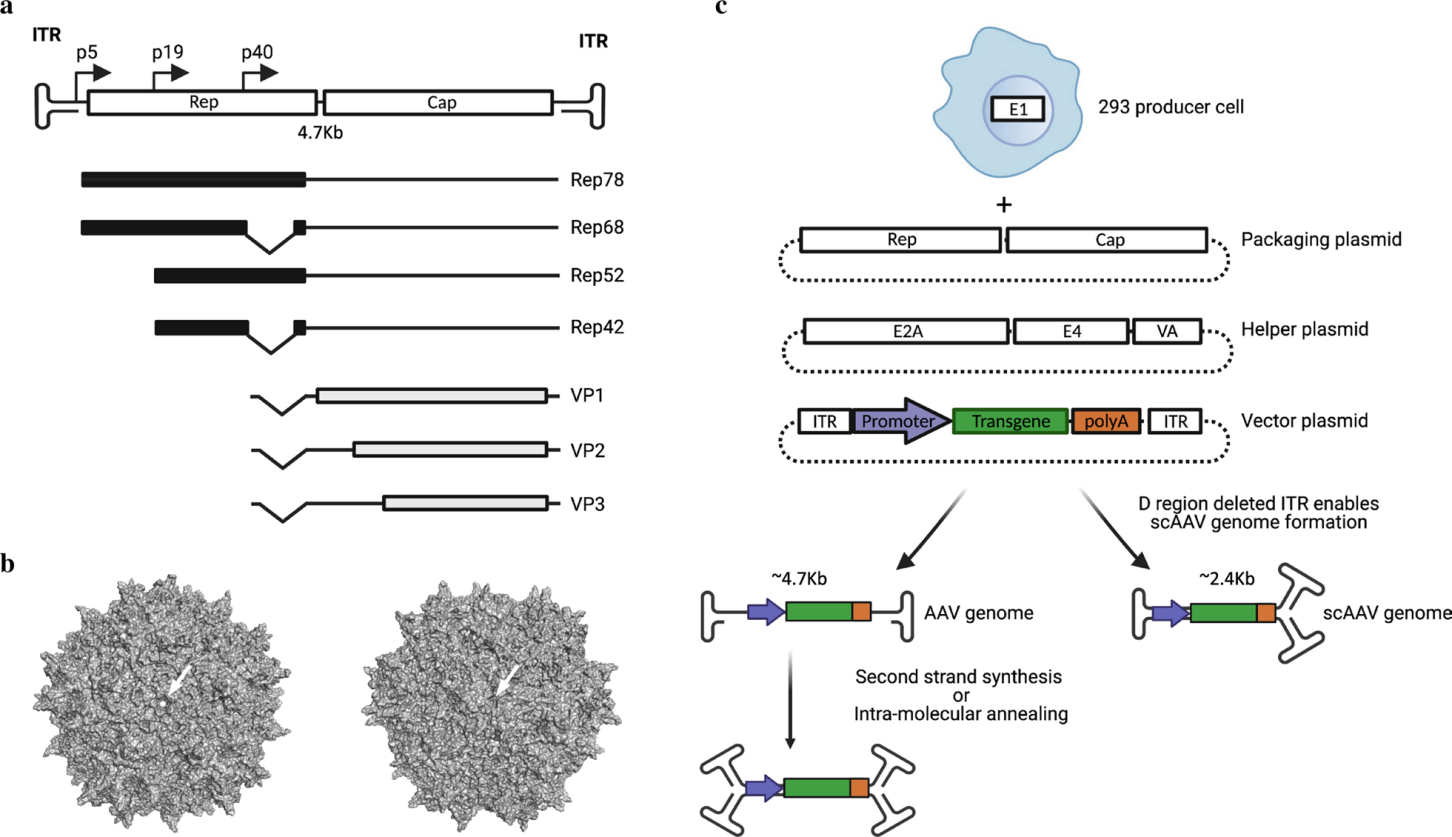
AAV vector biology. **a** genome organization of wild type AAV2 depicting its ssDNA genome and the 7 viral RNAs expressed from 2 genes Rep (Black boxes) and Cap (Grey boxes) and via the p5, p19 or p40 promoters. **b** crystal structure of the AAV2 virion (pdb: 1lp3) depicting the fivefold axis of symmetry (left, arrow) and threefold axis of symmetry (right, arrow). The AAV virion contains 60 VP proteins in a 1:1:10 ratio (VP1:VP2:VP3). **c** production of replication incompetent AAV vectors via transient plasmid transfection into 293 producer cells that express adenovirus type 5 E1 genes. Vectors containing standard or scAAV genomes can be generated following co-transfection of AAV packaging (AAV Rep and Cap containing), adenovirus type 5 helper (E2A, E4 and VA RNA expressing) and AAV vector plasmids without (standard) or with (scAAV) D region deletion in the left ITR

Over the last few decades, the development of adeno-associated virus (AAV) as a vector for gene delivery has advanced significantly [1, 2]. Recently, the AAV-based drugs Luxturna, a therapy to treat inherited blindness, and Zolgensma, a treatment for spinal muscular dystrophy, were approved by the FDA for use in the US, signifying important milestones for the establishment of AAV-based therapeutics in the clinic. The success of AAV as a gene delivery vector is due to several characteristics, including its nonpathogenic nature, its good safety profile, and its ease of production to clinical grade. Moreover, the minimal genome requirements of AAV to replicate permit replacement of most of the genome with foreign DNA, resulting in a packaging capacity of up to 4.7 kb in standard AAV vectors, or about half of that in self-complementary AAV vectors (Fig. 1). Importantly, AAV vectors display a broad species tropism that is malleable. So far, the approved AAV-based drugs and most clinical trials utilizing AAV vectors aim to supplement a defective gene with a new, working copy [3], but many studies have investigated AAV for the delivery of non-self therapeutic genes.

A promising application of AAV-based therapeutics is the delivery of gene-editing enzymes to correct defective genes [4]. In pre-clinical studies, several groups have reported the effective use of AAV-delivered CRISPR/Cas9 technologies to edit genes in animal models for diseases including Duchenne muscular dystrophy, hypercholesterolemia, and urea cycle disorders [5–9]. Similarly, the delivery of gene editing technologies to inactivate and eliminate viral reservoirs that enable persistent/chronic infections has recently gained substantial attention [10–14]. Indeed, recent reports of viral genome elimination using meganucleases and CRISPR/Cas9 in animal models for HSV and HIV chronic infection, respectively, support the use of AAV vectors as a treatment for chronic viral infections [10, 15, 16]. The AAV-mediated delivery of antiviral therapies is not limited to gene-editing enzymes. For example, several groups have used AAV vectors to deliver other antiviral therapeutics such as RNA inference molecules and virus-neutralizing antibodies [17, 18].

AAV-mediated delivery of curative antiviral therapeutics to sites of persistent viral infection requires much of the same vector optimization as traditional gene therapy, including promoter/transgene optimization for expression at therapeutic levels, efficient transduction of target cells, and limiting the immune response to the vector and transgene [19]. The simple premise underlying curative approaches to persistent viral disease is the delivery of virus-specific antiviral therapies to silence, mutate, or eliminate viral reservoirs within specific anatomical compartments. A high degree of precision is required on at least two levels to avoid off-target effects: specificity of target cell/tissue transduction and antiviral agent specificity for the virus and not the host. This review summarizes the strategies currently used to optimize AAV capsids, the principal determinant of vector tropism. We have focused on tissue-specific delivery of antiviral therapies targeting chronic/persistent viral infections in the liver, the peripheral nervous system, and T cells. These tissues/cells are important reservoir sites for numerous high impact human viral pathogens including hepatitis B virus (HBV), hepatitis C virus (HCV), Herpes simplex virus (HSV), Varicella Zoster Virus (VZV) and human immunodeficiency virus (HIV), all of which have a high public health burden.

AAV is a non-enveloped *Dependoparvovirus*, family Parvoviridae, with a capsid containing 60 subunits of three different proteins (VP1, VP2, and VP3) that have a common C-terminus and are produced via alternative translation of the AAV *cap* gene (Fig. 1). AAV cap is the major determinant of native AAV cell/tissue tropism and is thus a key modifiable genetic element to optimize transduction efficiency. Each capsid subunit has nine variable regions, peptide loops that protrude from the virion surface, that play a role in capsid assembly, genome packaging, cellular receptor interactions, and antigenic determinants for anti-capsid cellular and humoral immune responses [20].

Over 100 different AAV serotypes have now been isolated from humans, non-human primates (NHPs), and other species that exhibit a wide array of tissue and cell tropisms, determined mainly by the primary attachment receptor and co-receptor specificity of each serotype (Table 1). These naturally occurring, distinct serotypes have been widely screened for their ability to transduce many types of target cells and organs. Still, anti-AAV antibodies, which effectively neutralize AAV vectors and reduce transduction efficiency, are common in the human population and often cross-reactive, limiting the widespread clinical use of many serotypes clinically [21, 22]. Therefore, it is important to optimize the AAV capsid being used for each therapeutic application individually.

**Table 1:** Commonly used AAV vector capsids, their origin, receptor usage and in vivo tropism

AAV capsid	Naturally occuring	Likely species origin	Engineered	AAV Receptor usage	Other cellular receptor	Co-receptors	Tissue culture activity	In vivo tissue tropism
AAV1	Y	NHP	N	Y	Sialic acid		Moderate	**Skeletal muscle**, CNS, airway, retina, heart, liver
AAV2	Y	Human	N	Y	HSPG	FGFR-1, HGFR, αVβ1 and αVβ5 integrins, Laminin receptor, CD9	Good	Skeletal muscle, CNS, retina, liver
AAV3	Y	Human	N	Y	HSPG	FGFR-1, HGFR, Laminin receptor	Moderate	Skeletal muscle, liver
AAV4	Y	African green monkey	N	N	Sialic acid	Unknown	Poor	CNS, retina, kidney, lung
AAV5	Y	Human	N	Y	Sialic acid	PDGFR	Poor	Skeletal muscle, **CNS**, airway, retina
AAV6	Y	Human	N	Y	HSPG, Sialic acid	EGFR	Moderate	**Skeletal muscle**, airway, heart
AAV7	Y	Rhesus macaque	N	Unknown	Unknown	Unknown	Poor	Skeletal muscle, CNS, retina, liver
AAV8	Y	Rhesus macaque	N	Y	Unknown	Laminin receptor	Poor	**Skeletal muscle**, CNS, airway, retina, heart, **liver**
AAV9	Y	Human	N	Y	Galactose	Laminin receptor	Poor	**Skeletal muscl**e, **CNS**, airway, retina, heart, **liver**
AAV.rh10	Y	Rhesus macaque	N	Unknown	Unknown	Laminin receptor	Poor	Skeletal muscle, **CNS**, airway, retina, heart, **liver**
AAV.DJ	N	NA	Y	Unknown	HSPG	Unknown	Good	**Liver**, CNS, retina
AAV.LK03	N	NA	Y	Unknown	Unknown	Unknown	Poor	Human liver

Bold—used widely to target in vivo

*NHP* unknown non human primate, *NA* not applicable, *HSPG* Heparan sulfate proteoglycan, *FGFR* fibroblast growth factor receptor, *HGFR* hepatocyte growth factor receptor, *PDGFR* Platelet derived growth factor receptor, *EGFR* epidermal growth factor receptor, *CNS* Central nervous system

**Fig. 2:**
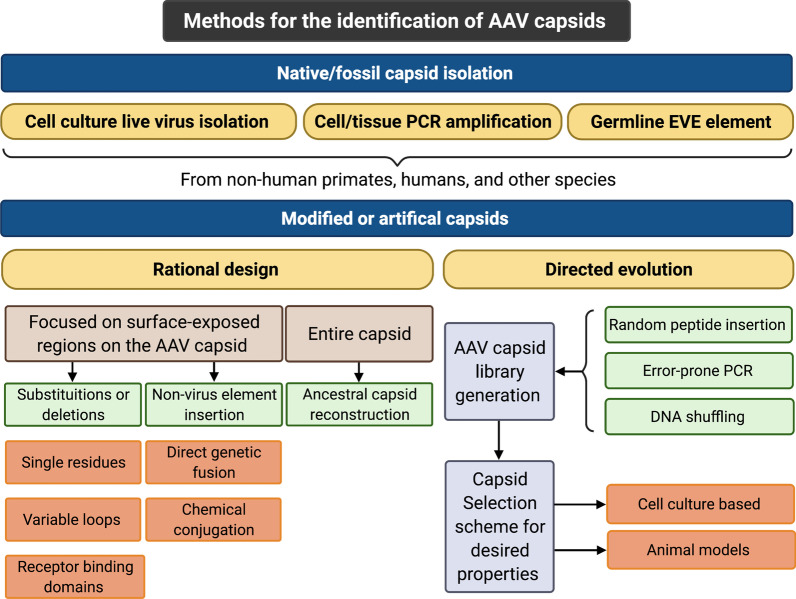
Methods for the identification of AAV capsids. The AAV capsid is the primary determinant of cell/tissue tropism. Efforts to identify novel capsids with enhanced target specificity and low immunogenicity are divided into two broad categories: native/fossil capsid isolation and capsid engineering (rational design or directed evolution). Native/fossil capsid isolation is achieved through live virus isolation from tissue culture, AAV-specific PCR or genome mining. AAV capsid engineering relies upon modification of key structural and genetic elements via rational design or directed evolution. Rational design exploits known aspects of AAV biology and structure, often focusing on surface-exposed regions of the AAV capsid. Directed evolution couples library-generated AAV capsid diversity and a selection scheme to identify variants with distinct properties. Strategies to generate AAV capsid libraries include error-prone PCR, DNA shuffling, and random peptide insertion. The libraries are then screened in cell culture systems, animal models, or a combination of both. Figure created with BioRender.com

**Fig. 3:**
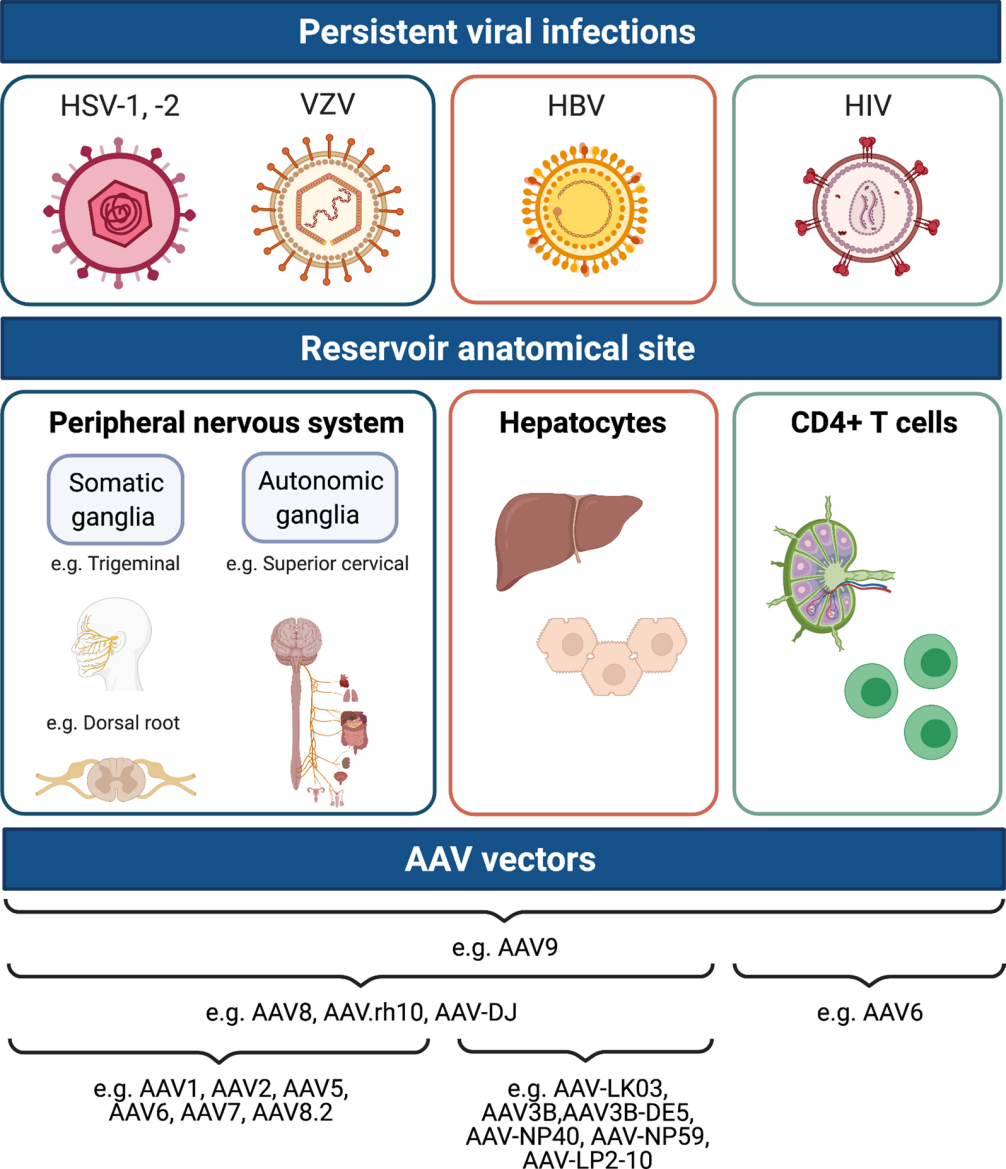
Identification of AAV variants with tropism for sites of persistent viral infections. The eradication or inactivation of viral reservoirs by direct delivery of virus-specific gene-editing enzymes or RNA-interference molecules represents a potentially curative strategy for persistent viral infections that currently affect billions of people worldwide. AAV is a promising delivery vector for these classes of antiviral therapy. Several AAV vectors discussed in this review and indicated below exhibit a high degree of tropism for the peripheral nervous system, liver, and CD4+ T cells, reservoir sites for Herpes simplex virus-1, 2 (HSV-1,2); Varicella Zoster virus (VZV); Hepatitis B virus (HBV); and Human immunodeficiency virus (HIV-1). Figure created with BioRender.com

Several strategies have been developed to optimize the AAV capsid for gene delivery, including rational design, directed evolution, and phylogenetic reconstruction of ancestral capsids (Fig. 2). These strategies offer several potential advantages that would be beneficial clinically, such as the ability to alter vector tropism, the ability to avoid vector sequestration in non-target tissues, the ability to reduce innate immune responses against the vector, the ability to evade pre-existing anti-vector humoral and cellular immune responses, and the ability to use lower effective AAV doses. Thus, engineering approaches that generate novel capsids derived from natural or artificial AAV capsids with optimized target tissue specificities and minimal antigenicity are an attractive solution to improve vector tropism for cells and tissues that harbor persistent viral infections (Fig. 3).

## Section I: Approaches to identify optimal AAV capsids

Since AAV1 was first identified in the 1960s as a contaminant of an adenovirus infected cell culture [21], many different AAV serotypes have been identified that display distinct yet overlapping tissue tropisms, underscoring the potential of AAV as a gene transfer tool for clinical use [23–25]. AAV is endemic and widespread in human populations. Thus, pre-existing humoral and cellular immunity presents a significant impediment to clinical use of AAV vectors, particularly when a human-derived serotype is used. Across numerous studies, the prevalence of anti-AAV neutralizing antibodies varies by serotype and geographical distribution. For example, AAV2 has the highest prevalence of anti-capsid antibodies [26–31]. Importantly, anti-AAV antibodies also exhibit a high degree of cross-reactivity [32], as seen in a recent longitudinal follow-up study of subjects who received intravascular AAV2-FIX in a clinical trial for severe hemophilia B. In this study, the participants had persistent multi-serotype cross-reactive neutralizing antibodies against the infused vector serotype AAV2, as well as serotypes AAV5 and AAV8, at up to 12–15 years post-vector administration [33]. These findings demonstrate that the presence of multi-serotype cross-reactive broadly neutralizing antibodies is a significant potential challenge to using AAV vectors in clinical applications as it limits the number of prospective patients that could benefit from AAV-based therapies. The long-lasting cross-reactive immunity also limits the ability to potentially re-administer a therapeutic via an alternative vector serotype. Thus, the search for new AAV serotypes that can evade host immune responses plus achieve high-level gene transfer in desired target cells and tissues at low vector doses continues.

### Natural AAV isolate screening

The first 6 identified AAV serotypes were isolated as live viruses from human or non-human primate (NHP) cell cultures and included AAV2, the first to be vectorized. AAV2 vectors can transduce many tissue types, including liver, muscle, lung, retina, and brain [3], and most early pre-clinical and clinical trials used AAV2 vectors to treat congenital diseases, including Leber's congenital amaurosis, retinal dystrophy caused by RPE-65 deficiency [33–36]. For example, in 2017, subretinal injection of the rAAV2-RPE65 became the first FDA-approved AAV-based gene therapy for a genetic disease [37]. More recently, the search for new serotypes with beneficial properties has resulted in PCR-based strategies that amplify AAV DNA from samples derived from humans, NHP, and other species [23, 24, 38]. To date, over a hundred variants have been isolated, mostly from humans and NHP, and many have been vectorized (Table 1). Notably, two of the first new serotypes isolated were the rhesus macaque-derived AAV8 and the human-derived AAV9 serotypes that have shown strong tropism for liver or multiple organs, respectively [39–41]. These serotypes have gained widespread clinical interest for use in diseases such as hemophilia B, where high FIX expression levels sufficient to elicit a phenotypic correction have been achieved using AAV8 vectors in hemophilia patients [42–50]. Ultimately, the isolation of new AAV serotypes with a wide range of tissue tropisms – primarily determined by different receptor and co-receptor usage [3, 28, 51, 52] – has significantly expanded the AAV vector tool kit with isolates that can transduce various tissues and partially evade neutralizing antibodies. This underscores the usefulness of screening for natural AAV across a wide swath of species. However, the isolation and characterization of novel variants is time-consuming, which has motivated the generation of novel engineered capsid mutants and library screening approaches to maximize the number of potential vector capsids that can target the tissue of interest.

### Directed evolution

Directed evolution has been widely used to identify new AAV capsids with beneficial properties via the screening of mutant capsid libraries, either in cell culture or in vivo. While the screening methods used have varied, to date three main approaches have been used to generate large AAV capsid libraries from which beneficial capsid variants are selected. The first approach to be used for AAV library generation relied on the observation that targeting peptides can be inserted into exposed surface loops of the AAV2 capsid at specific sites without altering capsid assembly [53]. Subsequently, it has been demonstrated that mutants generated by this screening approach can be retargeted to alternative cell types and evade antibody neutralization [53–56]. Libraries with random peptide insertions of 7–12 amino acids at AAV2 *cap* position 587/588 are now widely used to identify tissue-tropic mutant capsids. This approach has even been expanded to insert peptides at the same location in other serotypes such as AAV9 [57–62]. The second main approach used to generate AAV capsid libraries utilizes error-prone PCR of the AAV2 *cap* gene to create functional mutants and has been used to identify capsids with altered receptor binding affinity and the ability to evade neutralizing antibodies [63, 64]. Error-prone PCR has also been used to beneficially mutagenize other AAV serotypes. For example, surface-exposed residues found in the AAV9 *cap* gene were mutagenized via error-prone PCR, which enabled the identification of mutants with ablated tropism for liver and enhanced cardiac or musculoskeletal gene transfer [65]. The third main approach used to generate AAV capsid libraries pioneered DNA shuffling and PCR-mediated reassembly of randomly sheared AAV *cap* gene fragments from different AAV serotypes to generate capsid chimeras [66–69]. This DNA shuffling approach has also successfully identified novel capsids with altered tropism, the ability to evade neutralizing antibodies, or both. For example, AAV-DJ, a widely used chimera of serotypes 2, 8, and 9, was selected for its ability to evade neutralizing antibodies relative to other serotypes [66], and AAV-LK03, a chimera of 7 different serotypes, was selected for its ability to transduce human hepatocytes in humanized FRG mice [70], and is currently being evaluated in a clinical trial for hemophilia A [71]. In summary, directed evolution of AAV offers researchers the capability to select capsids from large and highly diverse mutant capsid libraries that provide distinct advantages over existing vectors, and has led to the identification of many new AAV capsids with beneficial properties such as improved transduction efficiency, reduced immunogenicity, broadened tissue tropism, or refined tissue tropism for specific cell populations [62, 65, 66, 72–77].

### Rational design

The utility of AAV as a gene therapy vector has fueled research to understand the basic biology of AAV. Sixty years after the discovery of AAV, structures for multiple AAV serotypes have now been resolved, allowing for a better understanding of capsid assembly and the basis for attachment to cell surface receptors [78–81]. This information, along with details about capsid receptor interacting domains, intracellular trafficking, uncoating, genome replication, and neutralizing epitopes, has informed the rational design of AAV vectors with enhanced properties through several different approaches. One approach to rational design relies on direct modification of the existing capsid amino acid footprint to transfer beneficial characteristics from one capsid to another. This approach may involve the substitution or deletion of single amino acids or entire domains between different AAV capsids. Examples of this approach include direct modification of single surface-exposed tyrosine residues to phenylalanine, preventing tyrosine-mediated ubiquitination and degradation of capsids after internalization [82]; substitution of variable loop domain residues to create a capsid with reduced immunogenicity [83]; or the substitution/deletion of an entire receptor binding domains that confer novel tropism upon the donor or recipient capsid [84]. Another approach to rational capsid design involves inserting non-virus elements into the capsid to convey beneficial properties. This has been primarily done to retarget AAV to alternate cellular receptors. For example, small targeting molecules have been linked to the AAV capsid via direct genetic fusion to AAV capsid proteins or via chemical conjugation to surface-displayed biotin acceptor peptides or split inteins. These studies have enabled AAV retargeting to cellular receptors including CD4, CD30, CD34, CD133, Her2/neu, and EpCAM, and offer great hope for developing AAV vectors with specificity for individual cell types [85–92]. A more recent approach to rational design has created entirely artificial AAV capsids using phylogenic analyses to reconstruct the gene sequences from common ancestors of extant AAVs. Reconstructed ancestral AAV capsids generated via this approach appear to be more thermostable and are highly potent for several different cell and tissue types [93, 94]. In a similar approach, germline endogenous viral elements (EVEs) from the *Dependoparvovirus* lineage have also been identified in a number of mammals, birds and marsupials [95–99]. These EVEs are now being used to guide the rational design of novel AAV vectors based on their distinct structural elements that could provide unique vector properties or tropisms [99]. As we continue to learn more about AAV and how it interacts with cells and tissues, new rational design strategies will be developed that enable us to discover improved vectors for different therapeutic applications.

## Section II: Identification of liver-tropic AAV vectors

### AAV-mediated delivery of antiviral therapies for chronic liver infections

Hepatitis B virus (HBV), hepatitis C virus (HCV), and hepatitis D virus (HDV) are hepatotropic viruses that cause viral hepatitis and can establish persistent/chronic infections in the liver. In 2015, an estimated 257 million people were living with chronic HBV infection, with an estimated 5% of these co-infected with the satellite HDV, an incomplete virus that requires HBV to establish infection and worsens HBV infection severity. A further 71 million people are estimated to have chronic HCV infection. It is estimated that approximately 4% of all new cancers are caused by chronic HBV and HCV infections [100], and together they were responsible for 1.34 million deaths in 2015 due to complications from cirrhosis and hepatocellular carcinoma (HCC), a figure projected to increase by > 60% in 2040 [101]. While a curative treatment has been recently developed for HCV, chronic HBV infection remains incurable, although viremia can be suppressed with antiviral drugs, and infection can be prevented by vaccination [100, 102–104].

Current antiviral treatments for HBV infection include nucleoside analogs (NAs) and immunomodulatory therapy, which can effectively suppress HBV replication, lower the rates of cirrhosis and HCC, and reduce the rate of mother-to-child transmission [105, 106]. However, these treatments require long-term adherence and rarely result in a permanent cure. The basis of HBV persistence is covalently closed circular DNA (cccDNA), the template for HBV genome replication and transcription in infected hepatocytes [107]. The cccDNA reservoir in hepatocytes is stable and can be maintained without the need for re-infection, as newly formed infectious particles can recycle from the cytoplasm back into the nucleus and replenish the reservoir [108]. Thus, elimination or inactivation of the cccDNA reservoir is the ultimate goal of new curative therapeutic strategies [12, 108–110].

Gene therapy to eliminate, mutate, or silence the HBV genome has been explored pre-clinically by a number of groups in several cell culture systems and animal models [reviewed elsewhere [12, 14, 109, 111]]. Some strategies include the delivery of RNA interference (RNAi) activators to inhibit HBV gene expression or the delivery of gene-editing enzymes such as CRISPR/Cas9, zinc finger nucleases, and transcription activator-like effector nucleases that cleave cccDNA, resulting in its degradation or inactivation through the introduction of mutations. Various delivery methods have been employed, including non-viral delivery using lipoplexes and adenoviral or lentiviral vectors. Of relevance to this review, recent studies have reported that AAV vectors can effectively deliver anti-HBV therapeutics to hepatocytes in vitro and the liver in vivo [18, 28, 111–116]. Therefore, AAV vectors are attractive gene transfer tools to deliver anti-HBV therapeutics due to their well-characterized liver transduction capabilities and safety profile.

In addition to the inherent potency and specificity of gene editing enzymes or RNAi activators for HBV, AAV vector optimization to maximize transduction and transgene expression in human hepatocytes is critical to gene therapy's effectiveness for viral infections. In recent years, significant advances in the development of liver-tropic AAV vectors have emerged from the gene therapy field that can also be harnessed to treat persistent hepatic infections [117]. Due to the liver's essential role in metabolism and systemic delivery of proteins into blood, many inherited and acquired diseases, such as hemophilia (A and B), α − 1 antitrypsin deficiency, and ornithine transcarbamylase deficiency, affect the liver and can potentially be corrected by liver-targeted gene therapy [2, 3, 72, 118]. For example, several clinical trials for hemophilia B have produced an array of data about AAV in humans, useful for improving hepatocyte tropism, transduction efficiency, and reducing immunogenicity [119]. Most of these studies have focused on engineering the AAV capsid, one of the major tropism determinants, through directed evolution, rational design, or a combination of both [120–122].

### Optimization of AAV vectors for liver-targeted applications

Recent efforts to improve AAV vectors for liver-targeted applications have focused on three areas: (1) maximizing human hepatocyte tropism, (2) evasion of neutralizing antibodies, and (3) streamlining pre-clinical evaluations of liver transduction efficiency for novel AAV capsids. The first two have mostly focused on engineering the AAV capsid, which largely determines both tropism and antigenicity. The third is motivated by discrepancies in transduction levels observed in clinical trials compared to those obtained in pre-clinical studies in cell culture and animal models. Over the last few years, liver humanized mouse models have emerged as an important tool to study AAV transduction differences between serotypes at the pre-clinical stage. To effectively study antiviral therapies against HBV, liver humanized mice are crucial since HBV cannot replicate in most small mammals. In these xenograft models, primary human hepatocytes (PHHs) are transplanted into immunodeficient mice with genetic mutations that elicit murine liver injury. This deficit confers a growth advantage to PHH over murine hepatocytes, enabling high engraftment levels [123–126]. Still, it is unclear how well these models recapitulate in vivo delivery in humans.

As previously discussed, AAV2 has successfully been used to treat blindness in clinical trials. In contrast, an AAV2 vector used for liver-targeted expression of FIX in early clinical trials for hemophilia B showed low efficacy, an interesting result given the robust transduction of human hepatocyte-derived cell lines by AAV2 vectors in vitro. A recent study suggests that tissue culture adaptations, possibly dating back to the early propagation of AAV2 in vitro, caused increased affinity for its primary cellular receptor heparan sulfate proteoglycan (HSPG). This resulted in enhanced transduction of primary hepatocytes and cell lines in vitro, while simultaneously reducing hepatocyte transduction in vivo [127]. In the same study, clade B isolates found in primary human liver samples that are similar to AAV2 showed reduced in vitro tropism for hepatocyte cells, but increased tropism for human hepatocytes in humanized FRG mice. Furthermore, these primary isolates could be adapted for tissue culture in Huh7 cells via iterative passaging with adenovirus type 5, but this resulted in attenuated in vivo transduction of hepatocytes. This study demonstrated the complexities of optimal capsid identification when using different in vitro or in vivo systems for selection.

The generation of AAV capsid libraries followed by a selective screen has significantly increased the repertoire of AAV capsids with modified transduction properties. In 2014, Lisowski et al. used the humanized liver FRG mouse model to identify AAV capsid mutants with improved human hepatocyte transduction in vivo [70]. This directed evolution methodology selected for variants with human hepatocyte receptor binding and entry capabilities. The group identified the capsid mutant AAV-LK03, which is closely related to AAV3B, differing only by eight amino acid changes. In an in vivo vector specificity analysis in humanized FRG mice, they showed that AAV-LK03 exhibits a stronger tropism for human hepatocytes in humanized mouse livers than AAV8, and AAV-LK03 is now being evaluated in a phase I/II clinical trial to treat hemophilia A (ClinicalTrials.gov: NCT03003533). Early results from this trial indicate that albeit safe at lower doses, high doses resulted in severe adverse events and subsequent loss of transgene expression in patients [71]. Recently, a rational design approach has been used to optimize AAV-LK03 for gene therapy applications via site-directed mutagenesis of surface-exposed residues. The site selection was informed by previous studies in AAV3B, which demonstrated that the elimination of specific surface-exposed serine and threonine residues enhances transduction efficiency while retaining viral tropism and cellular receptor interactions [128]. This study showed that applying rational design to library-derived variants is a promising tool for achieving superior results in clinical settings.

Since AAV-LK03 was first characterized, several studies have reported conflicting results regarding the superiority of AAV-LK03 over other liver-tropic serotypes in mice with human livers and NHP [114, 129–131]. In one comparative study of liver gene transfer using natural and engineered AAV serotypes, AAV3B, AAV8, AAVrh10, and AAV-LK03 all transduced NHP livers and human hepatocytes. In contrast to Lisowski et al., AAV-LK03 was not found to be superior to either AAV3B or AAV8 as a potent liver-specific vector [129]. More recently, the experimental variables that could affect AAV transduction of human hepatocytes were analyzed in a study using liver humanized FRG mice [132]. This study demonstrated that NTBC cycling, PHH donor origin, and the AAV vector dose could substantially affect transduction efficiency in human hepatocytes. These experimental variables could partially explain the discrepancies in AAV vector transduction efficiency seen between different laboratories using the FRG mouse and aid in the much-needed standardization of chimeric liver mouse models.

Although enhanced liver transduction efficiency has been seen with AAV-LK03 in some studies, this mutant is still moderately sensitive to pre-existing levels of neutralizing antibodies in humans [133]. To directly counteract humoral immunity against novel AAV capsids, a recent directed evolution study screened evolved human hepatotropic AAV capsids –obtained after five rounds of selection in FRG mice– against pools of human immunoglobulins pooled from thousands of patients to select capsids that can evade neutralization [120]. In this study, independent results from two laboratories showed that the newly identified capsid mutants AAV-NP40 and AAV-NP59 display superior transduction of human hepatocytes over AAV-LK03, regardless of human hepatocyte repopulation levels in liver humanized FRG mice. Importantly, reduced seroreactivity was seen for AAV-NP40 and AAV-NP59 relative to AAV-LK03 using serum from a cohort of 50 healthy US adults of mixed gender.

In another directed evolution approach, intravenous immunoglobulin was passively transferred into liver humanized mice before administering the AAV capsid library to identify mutants that could evade AAV neutralizing antibodies and transduce human hepatocytes in vivo [134]. After four cycles of selection, mutant AAV-LP2-10, composed of capsids derived from AAV2, AAV6, AAV8, and AAV9, was the dominant isolate. Using immunohistochemistry and flow cytometry as metrics for transduction efficiency in humanized mice, AAV-LP2-10 transduced human hepatocytes at similar levels to AAV8. Of note, several studies have reported AAV8 as a poor functional transducer of human hepatocytes in vivo [70, 135]. Nevertheless, AAV-LP2-10 was able to robustly escape pooled human immunoglobulins relative to AAV1, AAV2, AAV3, AAV6, AAV8, and AAV9. In twenty serum samples from the healthy donors, mutant AAV-LP2-10 had low neutralizing antibody titers, similar to AAV9 –one of the serotypes with the lowest prevalence of anti-AAV antibodies in healthy humans [31].

Individually, directed evolution and rational design have successfully created novel AAV vectors with enhanced liver-transduction capabilities and lower immunogenicity profiles. However, the combination of both methods has gained popularity in recent years [121, 134]. This approach involves the rational design of mutant libraries with targeted mutations in residues that are likely to affect capsid function during the screening process, thus maximizing the identification of mutants with desired features. A recent study combined rational design with directed evolution to select for liver-targeted AAV3B-derived variants [122]. The library design only allowed for randomization of residues in surface-exposed VRs while keeping the backbone sequence intact to maintain structural integrity. Moreover, to reduce the likelihood of detrimental amino acid substitutions, the allowed amino acids at each mutated site were limited to those that occur naturally at each residue in 150 native serotypes. The combinatorial AAV3B capsid library was serially screened for five rounds in vitro using 3D human hepatocellular carcinoma spheroid cultures. From this screen, variant capsid AAV3B-DE5, which contains 24 amino acid substitutions compared to AAV3B, became predominant. Although validation experiments in FRG mice demonstrated that AAV3B-DE5 transduces human hepatocytes at similar levels to AAV-LK03, the seroreactivity of AAV3B-DE5 relative to the parental AAV3B capsid improved significantly, showing that extensive changes in the amino acid sequence of VRs can effectively reduce pre-existing antibody neutralization without including neutralizing antibodies during the selection process.

In summary, the identification of liver-tropic AAV vectors in pre-clinical settings using animal models and cell culture systems has been extensively optimized to select for variants that can both transduce hepatocytes at high levels and escape pre-existing immunity. As the liver is an important target for gene delivery in many gene therapy applications, humanized liver mouse models will continue to be used in the search for vectors that meet both criteria.

## Section III: Identification of peripheral nervous system tropic AAV vectors

Neurons of the peripheral nervous system (PNS) have long been identified as key target cells in the study of pain. They are also major targets for treatment of the incurable and persistent alphaherpesviruses HSV-1, HSV-2, and varicella zoster virus (VZV), which are highly prevalent amongst humans [136–138]. After establishing an infection through peripheral mucosa, the respiratory tract or conjunctiva, human alphaherpesviruses all establish lifelong latent infections in neuronal cell bodies of sensory ganglia of the somatic or autonomic nervous system where their genomes persist in a latent episomal form that allows them to evade host immune responses and sporadically reactivate to initiate disease pathogenesis at peripheral sites.

The capability to deliver anti-viral therapeutics to sensory neurons in vivo or influence them via overexpression of genes or inhibitors would be extremely valuable, and AAV vectors have been identified as useful tools to accomplish this. As discussed below, multiple studies have shown that localized delivery of AAV vectors to individual ganglia results in efficient gene delivery to sensory neurons. Unfortunately, the small size of sensory ganglia limits the effectiveness of this approach on a large scale as both somatic and autonomic ganglia of the PNS are found throughout the body in multiple distinct locations so that comprehensive gene delivery would require many localized administrations. For example, there are 62 dorsal root ganglia (DRG) in humans, two for each of the 31 cervical, thoracic, lumbar, and sacral nerve pairs that emerge from each side of vertebrate along the spinal column. As an alternative to localized delivery, efforts have been made to identify natural or engineered AAV capsid variants that can facilitate gene delivery to multiple sensory ganglia when delivered through a single vector administration route that results in more widespread delivery. Here we review studies that have investigated gene transfer to sensory ganglia following local or systemic AAV vector delivery.

### Dorsal root ganglia

DRG houses neuronal cell bodies of the somatic nervous system that project from cervical, thoracic, lumbar, and sacral vertebrate and are involved in the relay of sensory information from peripheral sites to the central nervous system (CNS). DRG have been identified as a primary target for pain, spinal cord injury, and neurodegenerative disease studies. These clusters of neurons are also major 'reservoirs' for viral DNA during latent VZV and genital HSV infections [139–141], as alphaherpesvirus DNA can be detected in multiple DRG along the spine during latent infections. This makes DRG essential targets for curative antiviral therapies requiring gene transfer.

Localized AAV delivery routes such as direct lumbar or sciatic nerve injections can facilitate efficient gene transfer to individual or multiple closely situated DRG. Following localized injection directly into the DRG or the sciatic nerve, AAV vectors including serotypes 1, 2, 5, 6, 7, 8, 9, rh10, and an engineered AAV2-retro vector can all efficiently transduce neurons of the DRG in mouse, rat, and pig animal models [142–148]. In one of these studies, a head-to-head comparative analysis was performed in mice using seven different serotypes after localized delivery to the DRG. Serotypes 1, 5, and 6 performed best with up to 90% of neurons, both IB4+ and CGRP+, expressing the GFP transgene after 12 weeks [145]. Although localized delivery demonstrates the promise of AAV for DRG gene transfer, comprehensive gene delivery to multiple DRG situated along the spine will likely require a more generalized approach, so other studies have investigated widespread DRG gene delivery via different AAV delivery routes.

Indirect uptake via localized AAV delivery to afferent nerve terminals that project from peripheral sites has shown promise as an approach to transduce multiple ganglia. In one such study, AAV8-GFP vectors were injected into the footpad of mice, resulting in the transduction of > 90% of sensory neurons in multiple DRG that innervate the footpad [149]. Furthermore, efficient co-labeling of HSV+ neurons was seen from the AAV8-GFP vector, demonstrating the utility of this delivery approach in potential anti-HSV therapies. In a similar approach, an engineered AAV capsid vector (PHP.S) was delivered directly into the knee joint of mice, and uptake by nerves that innervate the knee from the DRG was seen, with up to 7% of lumbar L2-L5 neurons, including TRPV1+ neurons, transduced [150]. In the same study, delivery of therapeutic transgenes that target muscarinic receptors showed that it was possible to increase or decrease knee neuron excitability, thus demonstrating the use of AAV for studying the role of the DRG in pain responses. While the footpad and knee delivery routes show promise for DRG transduction in rodents, they may not translate well to large animal models. Other systemic delivery routes have also been studied as alternatives.

Delivery of therapeutic transgenes to multiple DRG via the cerebrospinal fluid (CSF) or blood offers a more straightforward route to widespread gene delivery in multiple DRG, and several groups have investigated this. When delivered via intrathecal (IT) injection, AAV vectors can efficiently transduce sensory neurons in multiple DRG [144, 147, 151–156] and AAV serotypes 5, 6, 8, 9 and rh10, as well as an engineered AAV8 capsid (AAV8.2) containing the AAV2 phospholipase A2 domain, have all shown efficient DRG transduction following IT delivery in mouse, rat, and macaque animal models. While these studies offer promise for future studies in humans, a pre-clinical study investigating gene transfer following IT delivery of an AAV9 vector expressing α-1-iduronidase (IDUA) to infant rhesus macaques offers the most hope for the future development of AAV-mediated antiviral therapies against alphaherpesviruses in humans [151]. This study showed that 4–29% of neurons within the cervical, thoracic, and lumbar DRG of 4 study animals expressed the IDUA transgene as long as three years and eight months post AAV administration.

Intravenous delivery of AAV vectors should, in theory, enable gene delivery to any cell within close proximity of the vasculature. However, unlike IT administration, IV delivery results in the circulation of AAV vectors through organs that are not part of the nervous system, such as the liver and spleen, where vector is often sequestered at high levels before it has a chance to see the target organ. Subsequently, sensory DRG likely see a lower relative effective AAV dose via IV delivery than IT, and mostly, AAV vectors do not appear to transduce sensory ganglia as efficiently through this administration route. Unfortunately, non-specific sequestration is inherent to most naturally occurring AAV capsids. However, one approach to prevent this has been to screen AAV capsid libraries for engineered variants that can transduce sensory ganglia when delivered IV to bypass non-specific sequestration. In vivo library selection has been used to identify AAV capsids with enhanced tropism for neurons of the CNS and PNS with great success and has yielded at least one novel capsid (AAV.PHP.S) that can efficiently transduce up to 82% of DRG neurons in mice after IV delivery [59, 61, 157]. While the PHP.S capsid does not appear to transduce other sensory ganglia as efficiently [10], future in vivo library selections may identify new AAV capsids that transduce DRG neurons and other sensory ganglia with high efficiency via any delivery route.

### Trigeminal ganglia

In mammals, two trigeminal ganglia (TG) sit below the brain, and house cell bodies of tactile, proprioceptive, and nociceptive afferent somatic sensory neurons that innervate the face. These TG are responsible for relaying craniofacial sensory and motor nervous stimuli from the left or right side of the face into the CNS through a large sensory root and small motor root that enters the brainstem. Like the DRG, the TG is a target for studies of pain, but it is also a reservoir for viral DNA during latent VZV and craniofacial HSV infection [139–141], and is a source of reactivating virus during cold sore or other head and neck HSV/VZV flare-ups. The TG is therefore an important target for curative anti-alphaherpesvirus gene therapies.

Direct injection of AAV delivery into the TG is extremely challenging due to its location immediately below the brain. Therefore, attempts to transduce sensory neurons of the TG using AAV vectors have mainly focused on indirect vector transductions methods involving delivery via projecting efferent neurons of the face or IV administration. Transduction of the TG has been attempted via delivery of AAV to the eye since efferent neurons from the TG project into the conjunctiva of the eye, but species-specific differences have been seen in delivery efficiency. In mice, the delivery of AAV to the eye has proved inefficient for TG delivery with minimal transduction of sensory neurons seen [158]. Conversely, in the rabbit, efficient transduction of TG neurons is seen following delivery to the eye, with AAV/HSV co-infection of sensory neurons also observed, demonstrating that this delivery approach could be used in the assessment of potential anti-HSV therapies [149]. As an alternative to the eye, intradermal AAV injection into the snout has been assessed in mice since efferent neurons project from the TG into the whisker pad. Following mouse whisker pad delivery, neuronal transduction in the TG can be seen from AAV1, AAV7, AAV8, or AAV9 vectors, with the highest levels (over 20%) seen with AAV1 [158]. Furthermore, it has been shown that gene editing meganucleases targeting HSV sequences can directly cleave HSV DNA in the TG of mice when AAV1 vectors are delivered via the whisker pad and a latent HSV infection is established via ocular challenge [159]. More recent efforts to target the TG have shown that IV administration of certain serotypes can provide more efficient transduction of the TG in mice. We have found that AAV8 and AAV.rh10 vectors can deliver transgenes to the TG with high efficiency when delivered IV [10]. Furthermore, when a triple serotype (AAV1/AAV8/AAV.rh10) combination HSV-specific meganuclease therapy is delivered IV, up to 55% of latent HSV-1 DNA found in the TG of mice during latent infections can be eliminated [10]. This data demonstrates the promise of AAV vectors for targeting the TG and also shows that the treatment/cure of persistent alphaherpesvirus infections may eventually be feasible.

### Autonomic sensory ganglia

The autonomic nervous system (ANS) is a branch of the PNS that unconsciously controls many bodily functions and can be divided into sympathetic 'fight-or-flight' neurons, parasympathetic 'rest-and-digest' neurons, and enteric neurons that control motor functions of the GI tract. The potential to transduce autonomic neurons would be useful for studying many aspects of neurological function across multiple body systems, and AAV vectors have been investigated to this end. Like somatic neurons, autonomic neurons of the sympathetic, parasympathetic, and enteric nervous systems can also harbor latent alphaherpesvirus DNA within neuronal cell bodies of distinct ganglia [160–162], so the ability to transduce autonomic neuronal populations is also important for the development of curative antiviral gene therapeutics targeting HSV and VZV.

While more limited in number than studies in the somatic nervous system, some studies have shown that ANS neurons can be efficiently targeted with AAV vectors. In mice, AAV8 and AAV9 vectors can efficiently transduce up to 57% of myenteric and submucosal neurons of the GI tract when delivered systemically via IV injection [163, 164]. In guinea pigs, AAV8 can transduce myenteric and submucosal gut neurons when delivered IV [165]. In cynomolgus macaques, AAV9 can transduce myenteric neurons of the stomach and small and large intestine after IV administration [165]. More recently, we were able to show that the neurons of the superior cervical ganglia (SCG), a part of the sympathetic ANS, can also be transduced with high efficiency by AAV vectors delivered IV. We found that AAV8 and AAV.rh10 vectors can transduce HSV infected neurons of the SCG in latently HSV-1 infected mice with high efficiency and that latent viral loads in the SCG can be reduced by more than 90% following triple serotype (AAV1/AAV8/AAV.rh10) HSV-specific meganuclease delivery [10]. Overall, these studies demonstrate the potential of AAV vectors for use in studying diseases of the autonomic nervous system.

### AAV-mediated toxicity in sensory ganglia

Despite the promise shown by AAV vectors for gene delivery to sensory neurons in vivo, several recent studies in large animals suggest that IV or IT delivery of high doses of AAV vectors may lead to asymptomatic injury to sensory neurons in vivo. Mononuclear cell infiltration and minimal to moderate asymptomatic degeneration of DRG neurons and associated axons has been seen in NHP, and more severe sensory neuronal lesions were seen in pig DRG [166–169]. While the toxicity seen in the DRG of NHP receiving AAV has been described as subclinical in some studies [151], a more comprehensive meta-analysis of 256 NHP receiving AAV vectors revealed that DRG pathology is almost always seen in NHP following AAV delivery as it was detected in 83% of animals receiving AAV via CSF delivery, and 32% of animals receiving AAV via IV injection. Furthermore, abnormal DRG pathology was shown to be independent of the different capsids (5), promoters (5), transgenes (20), or purification methods used for each AAV vector. These studies raise legitimate concerns for future studies that target sensory neurons of the PNS via AAV vectors. However, a recent study suggests that AAV-associated DRG toxicity can be reduced when a microRNA target is included in the vector backbone that selectively knocks down transgene expression in the DRG [170]. Further NHP studies addressing the mechanism and extent of adverse AAV-mediated pathology in DRG and other sensory ganglia will be needed as AAV vectors are advanced for use in therapies targeting the PNS.

## Section IV: Identification of CD4+ T cell tropic AAV vectors

CD4+ T lymphocytes are hematopoietic cells that play a major role in controlling infectious pathogens during the initiation of humoral and cellular immune responses by the host. Due to the many different and complex roles they play in host immune responses, CD4+ T cells have been identified as important targets in the study of autoimmune diseases, such as the severe combined immunodeficiencies (SCID), and for the development of cell-based immune-mediated therapeutics, such as chimeric antigen receptor (CAR) T cells. CD4+ T cells have also been identified as important targets for gene therapies targeting HIV, which selectively infects CD4+ T cells and CD4+ myeloid cells after infection and establishes lifelong infections via integration of its genome into the host chromosome. Unlike hepatocytes or sensory neurons, CD4+ T cells are ubiquitously found throughout the body in blood and other lymphoid and non-lymphoid tissues, which makes their transduction in vivo for the treatment of HIV highly challenging. This is of high importance for developing curative gene therapies against HIV that require widespread CD4+ T cell gene transfer to every infected cell throughout the body. Fortunately, despite the observed toxicity in the DRG, AAV vectors have generally shown a good safety profile when delivered systemically at high doses in several animal models, so they are being investigated as tools to deliver different types of therapeutics to CD4+ T cells both in vivo and ex vivo.

CD4+ T cells can be isolated from whole blood and transduced with gene therapeutics ex vivo before transplantation, or they can be directly targeted in vivo in blood and tissues when vectors are delivered systemically. For some therapeutic purposes, CD4+ T cells can also be targeted indirectly via transduction of hematopoietic progenitor cells that can differentiate into the lymphocyte lineage and become CD4+ T cells. AAV vectors have shown promise for all these approaches to T cell transduction, and AAV capsids showing promise for CD4+ T cell delivery have included naturally occurring capsids and capsids identified via directed evolution or rational design.

Initial attempts to determine whether naturally occurring AAV vectors could transduce hematopoietic cells identified AAV6 as a serotype that could efficiently transduce mouse myeloid cells [171]. Subsequent studies then demonstrated that AAV6 could efficiently transduce human CD34+ hematopoietic progenitor cells [172] and CD4+ T cells [173, 174], and AAV6 is now the vector of choice for many to deliver gene-editing enzymes or knock-in donors to primary T cells in the generation of CAR T cells or HIV receptor knockout CD4+ T cells ex vivo [173–177]. Gene editing of human CD34+ cells with AAV6 has also been shown to improve lymphopoiesis in a humanized mouse model of X-linked SCID (SCID-X1) that transplants ex vivo gene edited progenitor cells from SCID-X1 patients [178]. In an alternative approach, Smith et al. screened human hematopoietic cells for naturally occurring AAVs and identified 18 novel clade F AAV capsids that, when vectorized, were able to transduce hematopoietic progenitor cells that repopulated all lineages when transplanted in humanized mice [179]. While highly promising, a recent study suggests that these new isolates do not perform as well as AAV6 in human hematopoietic gene-editing studies [180], and AAV6 remains the current capsid of choice for ex vivo transduction of CD4+ T cells.

Approaches to identify capsids that transduce T cells using directed evolution or rational design have been few but show promise. In one study, AAV2 error-prone PCR, AAV2 loop insertion, and AAV shuffle libraries were pooled and screened for variants with improved transduction of the H9 T cell line [181]. Capsid variants with AAV2 loop peptide insertion were isolated after several rounds of screening that had increased transduction, demonstrating the utility of this approach. In two other studies, rational design was used to target either the AAV2 or the AAV6 capsid to CD4+ T cells via the surface display of small targeting molecules called direct ankyrin repeat proteins (DARPins) that have specificity for CD4 [88, 89]. These studies demonstrated that, when ablated of their native tropism, CD4-specific DARPin retargeted vectors can efficiently transduce CD4+ T cells both in vitro and in vivo. However, transduction of human CD4+ T cells was more efficient than rhesus macaque CD4+ T cells.

While efforts continue to identify better capsids that efficiently target CD4+ T cells, several recent animal studies have shown that systemic delivery of AAV-based therapeutics may offer a realistic pathway to effective anti-HIV therapies. Studies in HIV transgenic mice, humanized BLT mice infected with HIV, and SIV infected macaques have shown that AAV9 or AAV-DJ vectors expressing the *Sa*Cas9 gene in combination with sgRNAs that target the HIV or SIV long terminal repeat (LTR), and/or HIV *gag* can be used to excise portions of the HIV provirus in vivo when delivered systemically. HIV provirus excision was demonstrated in these studies in whole blood, CD4+ T cells, lymphoid, and non-lymphoid tissues throughout the body following a single AAV vector infusion [16, 182, 183], and up to 80% of SIV provirus DNA was lost from lymph nodes in SIV infected rhesus macaques that were pre-screened to be negative for exposure to AAV9 [16]. These studies suggest that with further work to identify new capsids with better in vivo tropism, effective AAV-mediated anti-HIV gene therapy could one day be a reality.

## Conclusions

Across the global human population, multiple persistent viral infections affect billions of people. While some of these infections are not associated with disease, or only cause disease in immunocompromised individuals, the proportion of viral infections that causes disease is of significant medical importance [184]. For instance, many persistent viral infections are risk factors for cancer development, accounting for 10–15% of all cancers [100, 185]. Although some are preventable by vaccination or treatable with drugs, as is the case for HBV, the permanent elimination of viral DNA from anatomical sites that sustain infection remains a challenge. Recent advances in gene-editing, antibody, and RNAi technologies represent promising therapeutic approaches to mutate or eliminate viral reservoirs. A fundamental aspect of these approaches is the localized and specific delivery of therapeutic agents to particular cell types, tissues, and organs. Recent advances and current challenges in developing AAV as a delivery vector for the treatment of monogenic, inherited diseases can inform the development and optimization of AAV as a vector for antiviral therapies to treat persistent infections (Fig. 3).

AAV vectors are a promising tool to achieve this because of their lack of pathogenicity, long-term transduction capacity, and relatively low immunogenicity, given their widespread natural prevalence in humans. As discussed here, the preclinical development of AAV vectors via capsid engineering in recent years has resulted in vectors with enhanced tissue tropism and potent transduction efficiencies in the liver, the peripheral nervous system, and CD4+ T cells, demonstrating the potential of AAV vectors to access anatomical sites harboring viral reservoirs. Nonetheless, significant challenges remain and have been best illustrated by studies in large animal models and clinical trial outcomes. A substantial obstacle for the implementation of AAVs as gene therapy vectors is the immunogenicity resulting from pre-existing anti-AAV antibodies, which many groups have undertaken preclinically by in vivo selection of AAV capsid libraries using directed evolution, rational design, or a combination of both. Other approaches to circumvent pre-existing immunity have explored AAV-specific plasmapheresis for the selective removal of circulating antibodies that can neutralize AAV vectors and the administration of immunosuppressive drugs before AAV administration. However, the effectiveness of these approaches has yet to be evaluated in humans [186–188].

The fast-paced preclinical development of novel AAV capsids for specific applications has significantly expanded the AAV catalog and holds promise for identifying vectors with desired properties for different applications. However, the extensive AAV ‘toolkit’ will require head-to-head comparisons of immunogenicity, biodistribution, and cell/tissue/organ transduction efficiencies to narrow down the best vectors to be evaluated clinically.

## Data Availability

Not applicable.

## Abbreviations

AAV: Adeno-associated viruses
HBV: Hepatitis B virus
HSV: Herpes simplex virus
HIV: Human immunodeficiency virus
NHP: Non-human primate
HCV: Hepatitis C virus
HDV: Hepatitis D virus
HCC: Hepatocellular carcinoma
cccDNA: Covalently closed circular DNA
RNAi: RNA interference
PHH: Primary human hepatocytes
HSPG: Heparan sulfate proteoglycan
PNS: Peripheral nervous system
VZV: Varicella zoster virus
DRG: Dorsal root ganglia
CNS: Central nervous system
CSF: Cerebrospinal fluid
IT: Intrathecal
IDUA: α-1-Iduronidase
TG: Trigeminal ganglia
ANS: Autonomic nervous system
SCG: Superior cervical ganglia
SCID: Severe combined immunodeficiencies
CAR: Chimeric antigen receptor
SCID-X1: X-linked SCID
DARPins: Direct ankyrin repeat proteins
LTR: Long terminal repeat
